# The Prevalence of Concha Bullosa and Nasal Septal Deviation and Their Relationship to Maxillary Sinusitis by Volumetric Tomography

**DOI:** 10.1155/2010/404982

**Published:** 2010-08-24

**Authors:** Kyle D. Smith, Paul C. Edwards, Tarnjit S. Saini, Neil S. Norton

**Affiliations:** ^1^Department of Oral Biology, Creighton University, 2500 California Plaza, Omaha, NE 68178, USA; ^2^Department of Periodontics and Oral Medicine, University of Michigan School of Dentistry, Ann Arbor, MI 48109, USA; ^3^Lieutenant Colonel, US Army DENTAC, Brooke Army Medical Center, Fort Sam Houston, San Antonio, TX 78234-5004, USA

## Abstract

The objective of this study was to determine the prevalence of concha bullosa and nasal septal deviation and their potential relationships to maxillary sinusitis. 883 CT scans taken at Creighton University School of Dentistry from 2005 to 2008 were retrospectively reviewed for the presence of concha bullosa, nasal septal deviation, and maxillary sinusitis. 67.5% of patients exhibited pneumatization of at least one concha, 19.4% of patients had a deviated septum, and 50.0% had mucosal thickening consistent with maxillary sinusitis. 49.3% of patients who had concha bullosa also had evidence of maxillary sinusitis. Only 19.5% of patients with concha bullosa also had nasal septal deviation, whereas 19.7% of patients with sinusitis also presented with nasal septal deviation. Although concha bullosa is a common occurrence in the nasal cavity, there did not appear to be a statistically significant relationship between the presence of concha bullosa or nasal septal deviation and maxillary sinusitis.

## 1. Introduction

With the recent widespread introduction of cone beam computed tomography (CBCT), dentists and otolaryngologists are better able to identify anatomical abnormalities and pathological states within the structures of the nasal cavity and the surrounding paranasal sinuses. Previously used radiographic techniques were frequently less effective at identifying irregularities in the sinuses [1]. Mucosal inflammation can be easily identified in computed tomography (CT) scans, arguably making this radiographic modality the standard for accurately evaluating the nasal cavity and paranasal sinuses [1]. 

On each side of the nasal cavity, there exists a superior, middle, and inferior concha. It is widely believed that osteomeatal obstructions may impede ventilation and mucociliary clearance from the sinuses, predisposing affected patients to sinus disease [1]. Less is understood about the role of a deviated septum or pneumatization of the conchae as potential contributors to the development of sinusitis [2]. While some studies suggest that deviations of the nasal septum or the presence of concha bullosa may interfere with proper airflow, potentially predisposing to sinus disease, other studies have produced contradictory findings [1, 3, 4]. The purpose of this study was to determine the prevalence of concha bullosa and nasal septal deviation and to examine their possible relationship to maxillary sinus disease.

## 2. Materials and Methods

A retrospective study was conducted of 883 CBCT scans taken between September 2005 and June 2008 at Creighton University School of Dentistry (Omaha, NE). This study was exempt from review by the Institutional Review Board. All scans were taken using an iCAT CBCT scanner (Imaging Sciences International) at a 0.3 mm voxel size. Scans were reconstructed using Osirix software and evaluated in the axial, sagittal, and coronal planes. Two trained investigators, well versed on the anatomy of the region, independently reviewed the scans. Any contradictory findings were reviewed by an anatomist. The gender and age of the patient were the only patient-specific variables included in this study.

Scans were reviewed for any nasal cavity and/or paranasal anatomical abnormalities, with specific evaluation on the presence of concha bullosa, deviated septa, and sinusitis of the maxillary sinuses. Concha bullosa was defined as the presence of pneumatization of any size within in the superior, middle, or inferior conchae. Septal deviation was defined as a deviation of greater than 4 mm from the midline. The presence of any radiographic mucosal thickening above the bony floor of the maxillary antrum was defined as abnormal [1, 4]. Data was analyzed with a Chi-square test using the SAS 9.1 program.

## 3. Results

Table 1 summarizes the age and gender distribution of the patient population examined. The mean age of the patients was 44.2 years of age, with a range of 4 to 99 years. Of the 883 scans evaluated, 43.6% were from male patients and 56.3% were female patients. 

**Figure 1:**
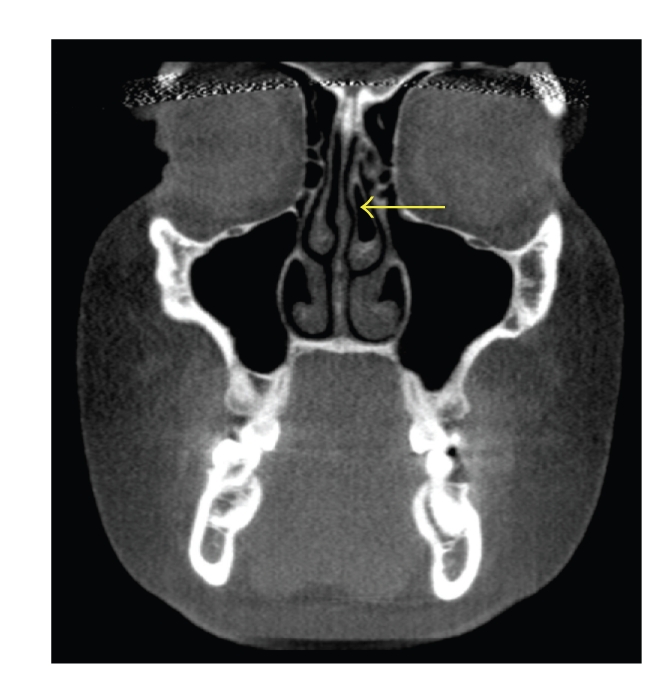
Coronal CT scan demonstrating the presence of left middle concha bullosa (arrow). No septal deviation or sinusitis is present. Note the size difference in the middle conchae, with the left middle concha larger than the right middle concha.

67.5% of the patient scans reviewed had evidence of pneumatization of the concha. From the 883 scans, 12.3% were located in one of the right conchae, 13.0% involving the left conchae, and 43.2% bilaterally distributed. The majority of concha bullosa were located in the middle concha; 7.8% on the right side, 8.3% left (Figure 1), and 20.8% bilateral. In the concha bullosa group, 56.3% were female and 43.7% were males (*P* = .856). The mean age of patients with concha bullosa (45.6 years of age) was similar to the overall study population (Table 2).

**Figure 2:**
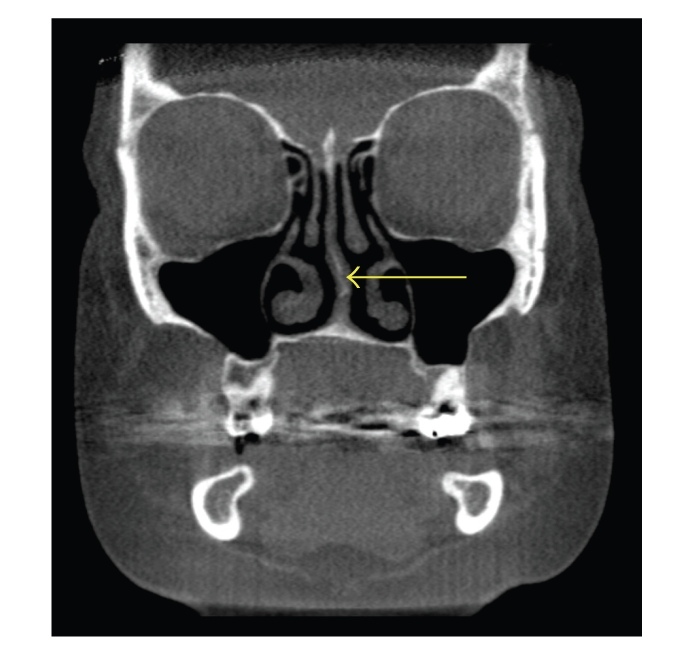
Coronal CT scan demonstrating left nasal septal deviation (arrow). No concha bullosa or sinusitis is evident.

19.4% of patients had deviated septa (Figure 2). There was no statistical difference between gender and the presence of nasal septal deviation (19.9% female; 18.9% were male; *P* = .703, Table 2). 

**Figure 3:**
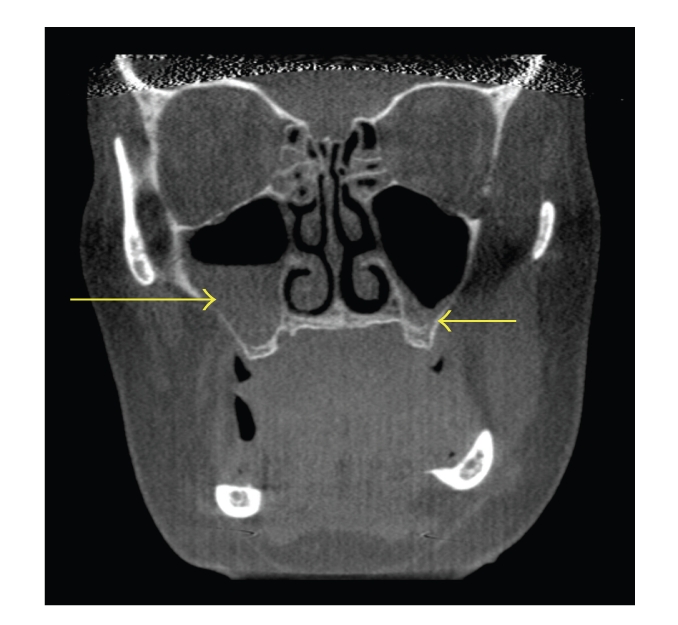
Coronal CT scan demonstrating bilateral maxillary sinusitis (arrows). The degree of sinus inflammation is more prominent in the right sinus. Concha bullosa or nasal septal deviation are not noted.

A total of 50.0% of patients had evidence of maxillary sinusitis. There was a statistically significant higher prevalence of maxillary sinusitis in males (61.8%) compared to females (41.8%; *P* < .0001). 12.1% had right maxillary sinusitis, 15.6% had left-sided involvement, and 21.0% had bilateral sinus disease (Figure 3). The mean age of patients with sinusitis was 44.3 (Table 2).

**Figure 4:**
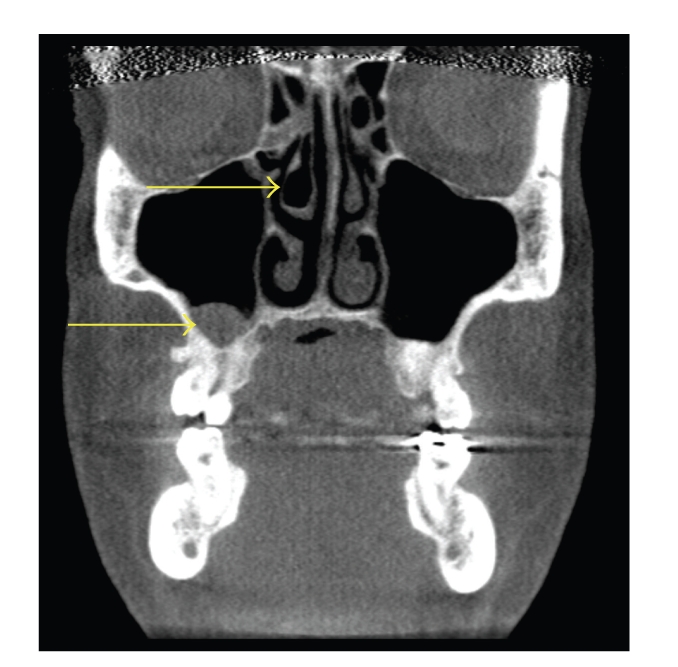
Coronal CT scan demonstrating right middle concha bullosa (superior arrow) and right maxillary sinusitis (inferior arrow). No nasal septal deviation is present. Note the difference in size of the right middle concha compared to the left middle concha.

**Figure 5:**
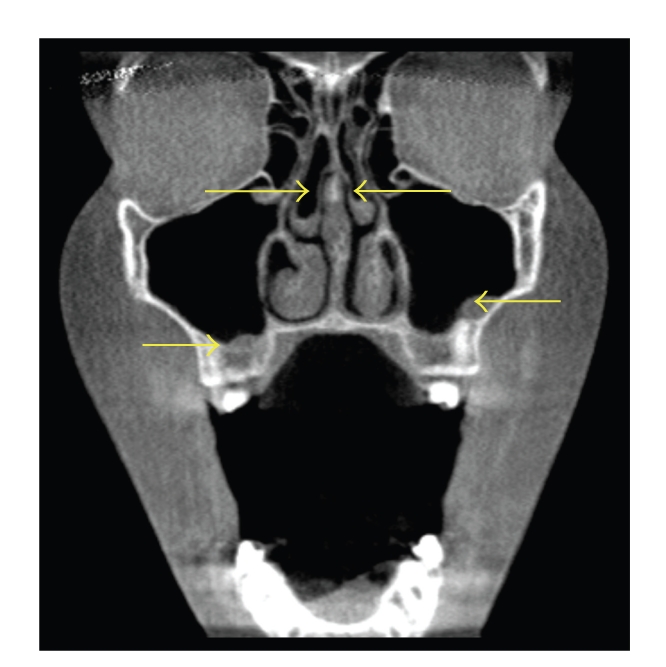
Coronal CT scans demonstrating bilateral middle concha bullosa (superior arrows) with bilateral maxillary sinusitis (inferior arrows). Note that there is more mucosal thickening on the left floor of the maxillary sinus than the right sinus floor, whereas the right concha bullosa demonstrates a greater degree of pneumatization compared to the left concha bullosa.

**Figure 6:**
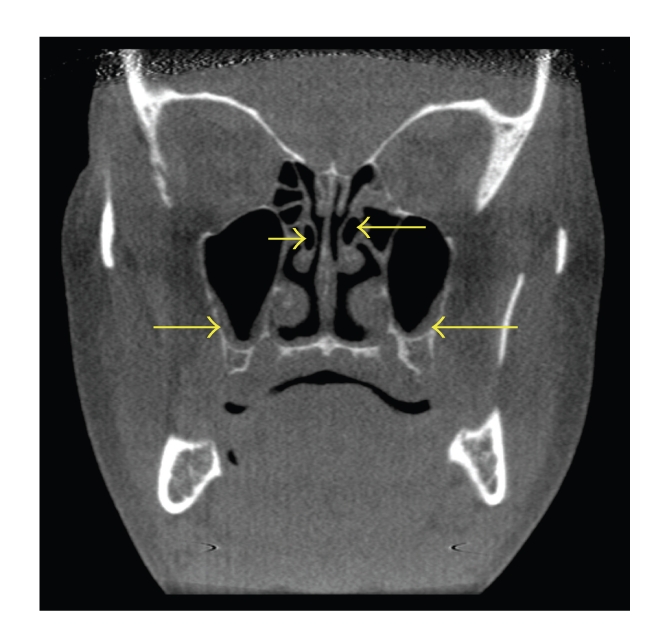
Coronal CT scan demonstrating bilateral middle concha bullosa (superior arrows) in combination with bilateral maxillary sinusitis (inferior arrows). Note the left concha bullosa (right superior arrow) is located slightly superior to the left concha. There is similar degree of sinus inflammation in both maxillary sinuses.

There was no statistical significance when comparing the relationship of patients with concha bullosa (67.6%) and those with sinusitis (41.8%). 49.3% of patients had a combination of both (Figures 4, 5, and 6), 50.7% had concha bullosa without evidence of sinusitis, and 33.5% had sinusitis in the absence of concha bullosa (*P* = .533, Table 3). 

The relationship between unilateral or bilateral concha bullosa and ipsilateral sinusitis was not statistically significant. Of the 109 patients with right concha bullosa, only 12.8% also had right maxillary sinusitis (*P* = .804). Of the 115 patients with left concha bullosa, only 18.3% of patients also demonstrated left maxillary sinusitis (*P* = .426). Of the 381 patients with bilateral concha bullosa, only 21.3% of patients had maxillary sinusitis (*P* = .559, Table 4). 

**Figure 7:**
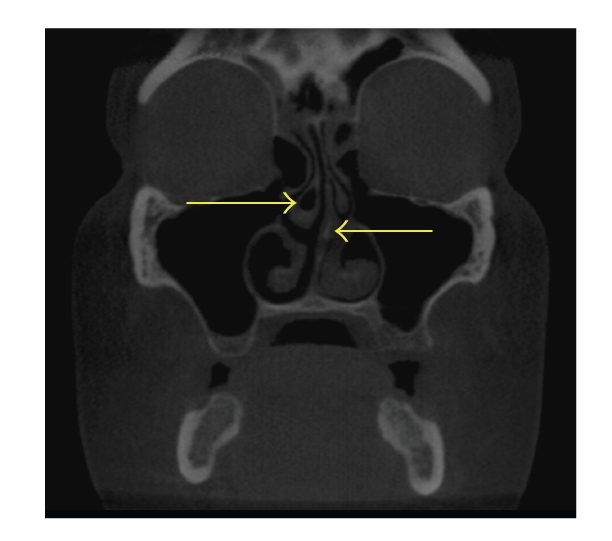
Coronal CT scan demonstrating right middle concha bullosa (left arrow) and left nasal septal deviation (right arrow). No sinus inflammation is present. Also note the differences in shape of the concha: the right middle concha is larger than the left middle; the left inferior concha is larger than the right inferior concha.

The relationship between the presence of concha bullosa and nasal septal deviation was not statistically significant. Of the 596 patients with concha bullosa, 19.5% also had deviation of the nasal septum (Figure 7). 80.5% of patients had concha bullosa without a deviated septum. 32.2% of the 171 patients with a deviated septum had no evidence of concha bullosa (*P* = .916; Table 5).

**Figure 8:**
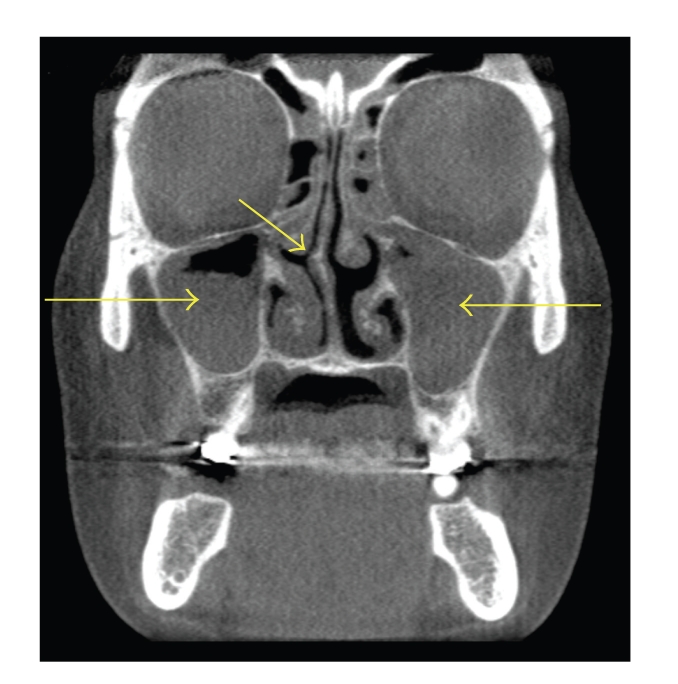
Coronal CT scan demonstrating right nasal septal deviation and severe bilateral maxillary sinusitis. No concha bullosa is present. The left maxillary sinus has a greater degree of inflammatory involvement than the right sinus.

Examining the potential relationship between sinusitis and nasal septal deviation, there was no statistical significance. 87 (19.7%) of the 442 patients with maxillary sinusitis also had nasal septal deviation (Figure 8). 355 (80.3%) of the patients with maxillary had no deviated septum. 84 (49.1%) of 171 patients with deviated septum had no evidence of maxillary sinus disease (*P* = .811; Table 6).

## 4. Discussion

In our study, 67.5% of patients had concha bullosa, which is somewhat higher than other studies, in which the prevalence of concha bullosa varied from 35% to 53% [1–4]. This variation may be due to differing criteria used to define concha bullosa. In our study, we defined any degree of pneumatization, regardless of size or location, as consistent with concha bullosa. Other studies restricted concha bullosa to specific locations on the turbinates and/or to a minimum size of pneumatization [1, 3, 4]. In Subramanian's study [4], there was a higher incidence of concha bullosa in females (58.9%) compared to males.

19.4% of patients in our study had nasal septal deviation, which is significantly lower than Stallman's 65% [3] and Sazgar's [2] 62.9% prevalences. The reason for this difference is most likely due to our stricter criteria for classification as deviated septum, which we defined as a deviation of greater than four millimeters from the midline. Stallman et al. [3] subjectively categorized deviations as mild, moderate, or severe, and Sazgar et al. [2] defined septal deviation as any asymmetric curvature of the septum.

Sinusitis, which was defined in our study as any evident thickening of the mucosa in the maxillary sinus, occurred in 50.0% of our patient population. Bolger's study [1] noted mucosal thickening of the sinus floor in 83.2% of patients. While the difference may be the result of referral bias (our patients were primarily referred for radiographic assessment prior to dental implant placement and not evaluation of suspected sinus disease), other potential variations such as seasonal bias, in which a small consecutive patient sample is chosen during a season that may predispose patients to higher incidence of allergies, may have contributed to this discrepancy. Our study was conducted over 2.5 years, spanning all seasons. One significant finding in our study was the relationship between sinusitis and gender, with males having a 20.0% higher incidence of sinusitis. Such a difference may be due to anatomical variations or mucosal secretion differences between the sexes.

While it has been suggested that abnormalities of the concha can predispose patients to obstruction of the sinuses, leading to chronic sinusitis [4–6], other studies with findings similar to those in the current study concluded that there was no correlation between the presence of concha bullosa and sinusitis [3, 5, 7]. Previous studies that supported the validity of a relationship have typically included a majority of patients with pre-existing chronic sinusitis [4].

While studies have suggested an association between septal deviation and the presence of concha bullosa [2, 3], the presence of septal deviations was usually associated with the presence of dominant or large concha bullosa [2, 3]. However, in our study, only 19.5% of patients with septal deviation had concha bullosa, suggesting that in many cases there is no relationship.

Regarding any potential relationship between nasal septal deviation and sinusitis, Hatipoglu et al. [8] found that there was an association between the degree of deviation and the presence of sinusitis. However, a meta-analysis conducted by Collet et al. [9] failed to confirm a definite relationship between these 2 factors, which is in agreement with the current study.

## 5. Conclusion

We found no definitive relationship between the presence of concha bullosa or nasal septal deviation and the development of maxillary sinusitis. 

## Figures and Tables

**Table 1:** Age distribution of the male and female population.

Age range (years)	Gender	
	Male	Female
1–10	9	12
11–20	49	46
21–30	83	75
31–40	40	34
41–50	41	86
51–60	70	107
61–70	55	77
71–80	22	36
81–90	11	13
91–100	0	1

Mean	42.8	46.7
Standard Deviation	20.2	19.7

**Table 2:** Prevalence and gender distribution of concha bullosa, nasal septal deviation, and sinusitis.

	Concha Bullosa		Nasal Septal Deviation		Sinusitis	
	Present	Absent	Present	Absent	Present	Absent
Total	596 (67.5%)	278 (31.4)	171 (19.4%)	712 (88.6%)	442 (50.0%)	441 (50.0%)

Gender						

Male	261 (68.3%)	121 (31.7%)	73 (18.9%)	310 (81.2%)	236 (61.8%)	146 (38.2%)
Female	334 (67.8%)	159 (32.3%)	98 (19.9%)	395 (80.1%)	206 (41.8%)	287 (58.2%)

**Table 3:** Relationship of concha bullosa and sinusitis.

		Concha Bullosa	
		Present	Absent
Sinusitis	Present	294 (49.3%)	148 (16.7%)
	Absent	302 (50.7%)	139 (15.7%)

**Table 4:** Relationship of right, left, or bilateral concha bullosa, compared to the presence of ipsilateral sinusitis.

Concha Bullosa	Ipsilateral Sinusitis present
Right	14/109 (12.8%)
Left	21/115 (18.3%)
Bilaleral	81/381 (21.3%)

**Table 5:** Relationship of concha bullosa and nasal septal deviation.

		Concha Bullosa	
		Present	Absent
Septal Deviation	Present	116 (19.5%)	55 (19.2%)
	Absent	480 (80.5%)	116 (19.5%)

**Table 6:** Relationship of concha bullosa and sinusitis.

		Concha Bullosa	
		Present	Absent
Sinusitis	Present	87 (19.7%)	355 (80.3%)
	Absent	84 (19.1%)	357 (80.95%)

## References

[1] Bolger WE, Butzin CA, Parsons DS (1991). Paranasal sinus bony anatomic variations and mucosal abnormalities: CT analysis for endoscopic sinus surgery. *Laryngoscope*.

[2] Sazgar AA, Massah J, Sadeghi M, Bagheri A, Rasool F (2008). The incidence of concha bullosa and the correlation with nasal septal deviation. *B-ENT*.

[3] Stallman JS, Lobo JN, Som PM (2004). The incidence of concha bullosa and its relationship to nasal septal deviation and paranasal sinus disease. *American Journal of Neuroradiology*.

[4] Subramanian S, Rampal GRL, Wong EFM, Mastura S, Razi A (2005). Concha bullosa in chronic sinusitis. *Medical Journal of Malaysia*.

[5] Nouraei SAR, Elisay AR, DiMarco A (2009). Variations in paranasal sinus anatomy: implications for the pathophysiology of chronic rhinosinusitis and safety of endoscopic sinus surgery. *Journal of Otolaryngology—Head & Neck Surgery*.

[6] Lee JS, Ko J, Kang HD, Lee HS (2008). Massive concha bullosa with secondary maxillary sinusitis. *Clinical and Experimental Otorhinolaryngology*.

[7] Zinreich SJ, Mattox DE, Kennedy DW, Chisholm HL, Diffley DM, Rosenbaum AE (1988). Concha bullosa: CT evaluation. *Journal of Computer Assisted Tomography*.

[8] Hatipoglu HG, Cetin MA, Yuksel E (2008). Nasal septal deviation and concha bullosa coexistence: CT evaluation. *B-ENT*.

[9] Collet S, Bertrand B, Cornu S, Eloy P, Rombaux P (2001). Is septal deviation a risk factor for chronic sinusitis? Review of literature. *Acta Oto-Rhino-Laryngologica Belgica*.

